# Predictive Modeling of Hypertension-Related Postpartum Readmission: Retrospective Cohort Analysis

**DOI:** 10.2196/48588

**Published:** 2024-09-13

**Authors:** Jinxin Tao, Ramsey G Larson, Yonatan Mintz, Oguzhan Alagoz, Kara K Hoppe

**Affiliations:** 1 Industrial and Systems Engineering University of Wisconsin Madison Madison, WI United States; 2 Department of Obstetrics and Gynecology MultiCare Rockwood Clinic Spokane, WA United States; 3 Department of Obstetrics and Gynecology, School of Medicine and Public Health University of Wisconsin Madison Madison, WI United States

**Keywords:** pregnancy, postpartum, hypertension, preeclampsia, blood pressure, hospital readmission, clinical calculator, healthcare cost, cost, cohort analysis, utilization, resources, labor, women, risk, readmission, cohort, hospital, statistical model, retrospective cohort study, predict, risk

## Abstract

**Background:**

Hypertension is the most common reason for postpartum hospital readmission. Better prediction of postpartum readmission will improve the health care of patients. These models will allow better use of resources and decrease health care costs.

**Objective:**

This study aimed to evaluate clinical predictors of postpartum readmission for hypertension using a novel machine learning (ML) model that can effectively predict readmissions and balance treatment costs. We examined whether blood pressure and other measures during labor, not just postpartum measures, would be important predictors of readmission.

**Methods:**

We conducted a retrospective cohort study from the PeriData website data set from a single midwestern academic center of all women who delivered from 2009 to 2018. This study consists of 2 data sets; 1 spanning the years 2009-2015 and the other spanning the years 2016-2018. A total of 47 clinical and demographic variables were collected including blood pressure measurements during labor and post partum, laboratory values, and medication administration. Hospital readmissions were verified by patient chart review. In total, 32,645 were considered in the study. For our analysis, we trained several cost-sensitive ML models to predict the primary outcome of hypertension-related postpartum readmission within 42 days post partum. Models were evaluated using cross-validation and on independent data sets (models trained on data from 2009 to 2015 were validated on the data from 2016 to 2018). To assess clinical viability, a cost analysis of the models was performed to see how their recommendations could affect treatment costs.

**Results:**

Of the 32,645 patients included in the study, 170 were readmitted due to a hypertension-related diagnosis. A cost-sensitive random forest method was found to be the most effective with a balanced accuracy of 76.61% for predicting readmission. Using a feature importance and area under the curve analysis, the most important variables for predicting readmission were blood pressures in labor and 24-48 hours post partum increasing the area under the curve of the model from 0.69 (SD 0.06) to 0.81 (SD 0.06), (*P*=.05). Cost analysis showed that the resulting model could have reduced associated readmission costs by US $6000 against comparable models with similar *F*_1_-score and balanced accuracy. The most effective model was then implemented as a risk calculator that is publicly available. The code for this calculator and the model is also publicly available at a GitHub repository.

**Conclusions:**

Blood pressure measurements during labor through 48 hours post partum can be combined with other variables to predict women at risk for postpartum readmission. Using ML techniques in conjunction with these data have the potential to improve health outcomes and reduce associated costs. The use of the calculator can greatly assist clinicians in providing care to patients and improve medical decision-making.

## Introduction

Hypertensive disorders of pregnancy (HDP) are common and estimated to occur in 10% of pregnancies in the United States. In addition to complicating the management of pre- and postdelivery periods, hypertension is the leading cause of postpartum readmission, accounting for 9.3%-27% of postpartum readmissions [1-3]. Postpartum readmission is costly, both in health care dollars and in quality-of-life measures for mothers and new families. In addition, HDP increases maternal morbidity and mortality and is associated with an increased risk of cardiovascular disease later in life [4-8].

A study published in the *Journal of Hypertension* in 2018 estimated that preventable postpartum readmission in women with hypertension resulted in 20,240 excess inpatient hospital days and US $36 million excess medical costs [9]. Rates and reasons for readmission have been under recent scrutiny and offer an area to improve health care delivery and preventative care. All-cause hospital readmission rates are on the rise with risk factors for all-cause postpartum readmission including public insurance, race, presence of comorbid conditions including hypertension and diabetes, and cesarean section [3].

Approximately 30% of women who experience hypertension-related postpartum readmissions do not have antecedent diagnoses of hypertension, thus making it imperative to include normotensive patients without an HDP before postpartum discharge in evaluating for postpartum readmission [10]. As such, our objective was to identify key clinical variables, in addition to demographic characteristics, implicated in postpartum readmission of all birthing persons using a machine learning (ML) model. This prediction task is challenging because while costs related to readmission are high, readmission rates are low resulting in highly imbalanced data sets that are challenging to use in training ML models. For instance, while existing models have strong overall accuracy performance (out of sample, an area under the curve of 0.81) [11], they do so by trading off high specificity for low sensitivity which could result in many readmission cases going undetected and not properly treated. We hypothesized that blood pressure metrics during labor, not just post partum, would impact readmission rates. Similarly, we hypothesized that antihypertensive medication administration and high preeclampsia laboratory values during initial readmission would increase the readmission rate.

## Methods

### Ethical Considerations

We obtained institutional review board approval (#2016-006). Individual patient consent was not required due to the retrospective study design. The data set was deidentified before study analysis. No compensation was provided to human participants as this was a retrospective study that involved development of a retrospective data set using electronic medical records (EMRs).

### Chart Review and Inclusion

We initially performed a retrospective chart review of all patients who delivered at a single, midwestern academic center hospital between 2009 and 2015. Inclusion criteria for this study included all women who delivered a baby in this time frame. We wanted to ensure that we captured all hypertension-related readmissions within 42 days post partum regardless of a diagnosis of hypertension before hospital discharge from the delivery of their infant. The primary outcome was hypertension-related readmission; therefore, all readmissions included in this data set were specific to hypertension only. To confirm our previous results and create a larger sample size, we extended the study population to include births from 2016 to 2018 and used a similar process to manage the data of all patients who delivered at the same birthing hospital. We used the hospital’s PeriData website [12] data set, which is used to contribute birth-related outcomes to the state-wide database for clinical perinatal information and additional hospital-run reports to obtain additional data available from the EMR. We collected demographic as well as clinical data, including blood pressure measurements during labor and post partum, laboratory data, and medication administration at the patient level to be our predictor variables. Hospital readmissions and our prediction response variable were verified by patient chart review. Given that the data came from multiple sources and had missing observations, the raw data set could not be used directly for analysis.

### Analytics Plan

#### Data Processing and Feature Engineering

We processed the raw data set and then merged the processed data including patient demographics, blood pressure measurements, medication administration, and laboratory information from different sources into 1 pandas data frame [13]. Race and ethnicity were entered in the medical chart based on the patient’s self-identity at the time of admission to the health care system. Laboratory results were included in this analysis because they are involved in the classification and severity of HDP. Laboratory results included liver function tests, hemoglobin and platelet counts, creatinine, and urine protein. We analyzed blood pressure records with timestamps and identified the highest systolic blood pressure and associated diastolic blood pressure during 3 time periods, that are, labor, 0-24 hours post partum, and 24-48 hours post partum, because we expected blood pressure during labor and post partum to be important features for predicting hypertensive readmissions. Using the medication administration data from the EMR, we constructed the following binary (yes or no) attributes for the following medication name and route administered: (1) oral labetalol, (2) intravenous labetalol, (3) oral nifedipine-immediate release, (4) oral nifedipine-extended release, (5) intravenous hydralazine, and (6) oral ibuprofen. To obtain these features, we started with a full medication data set for each patient’s medical registration number that included the medication name, time administered, dosage, and route of administration. This meant that there were multiple entries per medical registration number if a particular patient was given that medication more than once. The key challenge of using the medication data was that there were significant missing data; moreover, not all patients received all medications, and the individual medication schedules could be infrequent. For this reason, we considered only the binary attributes instead of the full medication schedule to ensure that the data points were dense enough for the analysis.

#### Predictive Model Training and Validation

We used a cost-sensitive random forest method to predict which patient would experience a hypertension-related postpartum readmission [14]. Since the data set was imbalanced (only 170 readmissions out of 32,645 participants), the use of class weights that penalize false negatives significantly higher than false positives was necessary to avoid ML models that predict every sample as the negative class. We considered other candidate classifiers namely logistic regression with L1 or L2 regularization, support vector machines (SVM) with polynomial, radial basis function or sigmoid kernel, and a standard decision tree approach for the prediction task. To measure the predictive performance of each model, we considered a combination of different metrics. In the case of imbalanced data, reporting high accuracy may be inappropriate since a highly accurate model could simply ignore the rare class and still achieve high accuracy. Therefore, we considered 2 complementary scores for assessing our model namely balanced accuracy and the *F*_1_-score [15]. The balanced accuracy can be thought of as balancing the frequency of true positives and true negatives. When calculating accuracy, it can be calculated by averaging the true negative rate (specificity) and the true positive rate (sensitivity) of the model. In addition to prediction accuracy, since our setting has a low frequency of positive cases, we needed to ensure our selected model had high precision (alternatively low false alarm rate). For that reason, we also considered the *F*_1_-score, which measures the balance between the precision and the true positive rate. We tuned the hyperparameters of each model using cross-validation. For the outer loop, we iterated every hyperparameter combination. Then we performed stratified 5-fold cross-validation in the inner loop and optimized the hyperparameters by evaluating the average balanced accuracy. Each model was trained using its respective classifier implementation from scikit-learn [16].

We trained models on the 2009-2015 data and 2016-2018 data individually and validated them using the 5-fold cross-validation pipeline. The purpose of this was to see if different factors impacted readmission rates and decisions between the 2 time periods. For added validation, we computed the performance of models trained on the 2009-2015 data set using the 2016-2018 data to evaluate our pipeline. The final model deployed in practice was tuned using the combined data set and 5-fold cross-validation. We performed a feature importance analysis on the best models chosen by cross-validation for each data set.

#### Cost Analysis and Estimating Clinical Impact

To estimate the clinical impact of predictions, we completed 2 different forms of cost analysis. For each candidate model considered, we used the above cross-validation procedure to compute their estimated implementation costs.

We estimated the value of a false negative (an unplanned or unpredicted readmission) to be US $20,439 and the value of a false positive (the price of labetalol for 6 weeks for a patient who ultimately did not need it) to be US $36. These costs were based on estimates derived from our previous research [17]. In addition, for the cost-sensitive random forest model (which we ultimately determined was the most effective model), we performed an additional analysis. For this analysis, we took the model’s score for how likely a patient was to be classified as needing readmission and compared it with a predictive threshold. If the model score was larger than the threshold, the model would predict that the patient would be readmitted. When the threshold is <0.5, more patients are predicted to be readmitted and if the threshold is >0.5, more patients are predicted to not be readmitted. We used leave-one-out cross-validation to compute the overall medical costs and balanced accuracy for different thresholds between 0 and 1. The goal of this analysis was to see how model scores should be interpreted in practice by decision makers so that overall medical costs are minimized.

## Results

### Data Overview

From January 2009 to December 2018, a total of 39,133 women delivered at our hospital; however, only 32,645 had complete medical records available for analysis. Of these, 170 women were readmitted for a hypertension-related diagnosis. There was a statistically significant difference between the readmitted group and the not readmitted group in terms of maternal age, gestational age at delivery, race, BMI, mode of delivery, and hypertension diagnosis. The readmitted group was more likely to be older, having earlier gestational age at delivery, Black race, higher BMI, cesarean delivery, and having a diagnosis of chronic or pregnancy-induced hypertension (Table 1). The rate of hypertension diagnosis in our sample was 9%. The rate of readmission was 0.5%.

**Table 1:** Patient demographics and comparisons between the readmitted group and the not readmitted group.

Characteristics				All patients (N=32,645)		Readmitted (n=170)		Not readmitted (n=32,475)		*P* value	
Maternal age, mean (SD)				30.5 (5.3)		32.9 (5.7)		30.5 (5.3)		<.001	
**Parity, n (%)**											.29
	Nulliparous		10,786 (33)		61 (35.9)		10,725 (33)				
	Multiparous		17,946 (55)		95 (55.9)		17,851 (55)				
	Unknown		3913 (12)		14 (8.2)		3899 (12)				
Gestational age at delivery in weeks, mean (SD)				38.9 (2.4)		37.7 (2.5)		38.9 (2.4)		<.001	
**Race, n (%)**											<.001
	White		26,188 (80.2)		130 (76.5)		26,058 (80.2)				
	Black		1221 (3.7)		18 (10.6)		1203 (3.7)				
	Asian Indian		2532 (7.8)		12 (7.1)		2520 (7.8)				
	Asian, other		855 (2.6)		3 (1.8)		852 (2.6)				
	American Indian or Native		457 (1.4)		1 (0.6)		456 (1.4)				
	Native Hawaiian		67 (0.2)		0 (0)		67 (0.2)				
	Unknown or other		1325 (4.1)		6 (3.5)		1319 (4.1)				
**Hispanic, n (%)**											.05
	Yes		2937 (9)		8 (4.7)		2929 (9)				
	No		29,708 (91)		162 (95.3)		29,546 (91)				
BMI^a^, mean (SD)				26.5 (8.6)		28.7 (8.6)		26.5 (8.9)		.001	
**Mode of delivery, n (%)**											<.001
	Vaginal		20,217 (61.9)		76 (44.7)		20,141 (62)				
	Vaginal vacuum		1695 (5.2)		8 (4.7)		1687 (5.2)				
	Vaginal forceps		582 (1.8)		3 (1.8)		579 (1.8)				
	Cesarean section		10,151 (31.1)		83 (48.8)		10,068 (31)				
**Hypertension diagnosis, n (%)**				2952 (9)		96 (56.5)		2856 (8.8)		<.001	
	**Chronic hypertension**										
		Without preeclampsia	299 (1)		16 (9.4)		283 (0.9)				
		With preeclampsia	284 (0.9)		15 (8.8)		269 (0.8)				
	Gestational hypertension		793 (2.4)		13 (7.6)		780 (2.4)				
	**Preeclampsia**										
		Mild	582 (1.8)		26 (15.3)		556 (1.7)				
		Severe	828 (2.5)		18 (10.6)		810 (2.5)				
	Unspecified		166 (0.5)		8 (4.7)		158 (0.5)				

^a^BMI: weight in kilograms divided by the square of height in meters.

### Predictive Model Results

**Figure 1:**
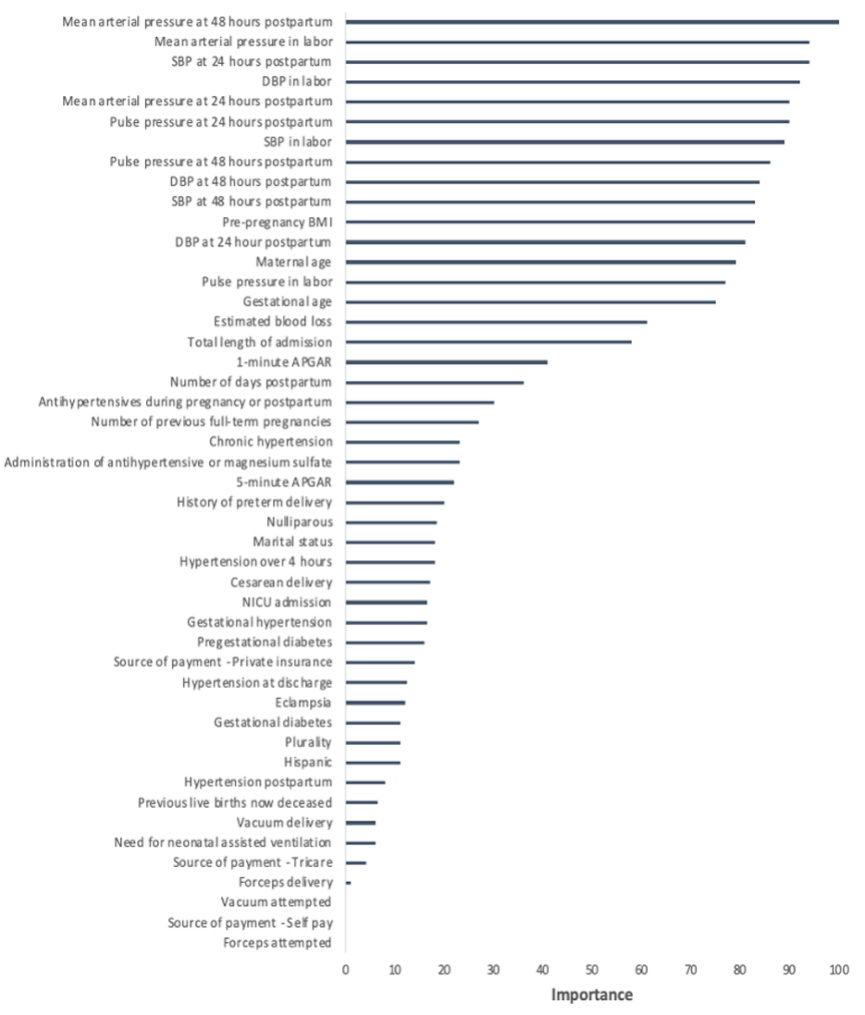
Feature importance plot for hypertension-related postpartum readmission, 2009-2015. APGAR: appearance, pulse, grimace response, activity, respiration; DBP: diastolic blood pressure; NICU: neonatal intensive care unit; SBP: systolic blood pressure.

During our initial analysis of the data from 2009 to 2015, we evaluated 47 clinical and demographic variables to assess their importance in predicting postpartum readmission (Figure 1). The variables most important for predicting readmission included blood pressure parameters during labor and through the postpartum period as well as factors such as prepregnancy BMI, maternal age, and gestational age at delivery. Variables that had less predictive value included an HDP, administration of antihypertensive medication, and mode of delivery. To increase the predictive accuracy of the model, many of these variables were excluded from the next analysis. Even with fewer variables, again the diagnosis of HDP and mode of delivery were of least importance and blood pressure data during labor and post partum were most important. Additional details on the model feature importance and feature correlations can be found in the Multimedia Appendix 1 [18]. Through cross-validation analysis, we found that the best model in terms of balanced accuracy and *F*_1_-score was the random forest model. We performed an additional validation by using the best-tuned models from the 2009 to 2015 data set on the 2016 to 2018 data sets; the results are shown in Table 2. Each model was trained only on the 2009-2015 data and was used to predict readmission for patients in the 2016-2018 data. As shown in Table 2, the random forest model achieves the best-balanced accuracy and *F*_1_-scores among all candidate models. Please refer to the Multimedia Appendix 1 [18] for the full set of cross-validation parameters for each model.

**Table 2:** Predictive performance of models trained using the 2009-2015 data set on the 2016 to 2018 data sets.

Model type	Specificity, %	Sensitivity, %	Precision or PPV^a^, %	NPV^b^, %	*F*_1_-score	Balanced accuracy, %	Cost (US $)
Random forest	70.86	75.81	1.38	99.82	0.027	73.33	426,240
Decision tree	64.94	75.81	1.15	99.8	0.023	70.37	450,828
SVM^c^	53.21	90.32	1.03	99.9	0.020	71.77	316,512
Logistic regression L1	62.12	83.87	1.17	99.86	0.023	72.99	360,864
Logistic regression L2	63.09	83.87	1.21	99.86	0.024	73.48	356,832

^a^PPV: positive predictive value.

^b^NPV: negative predictive value.

^c^SVM: support vector machines.

### Feature Importance Analysis

**Figure 2:**
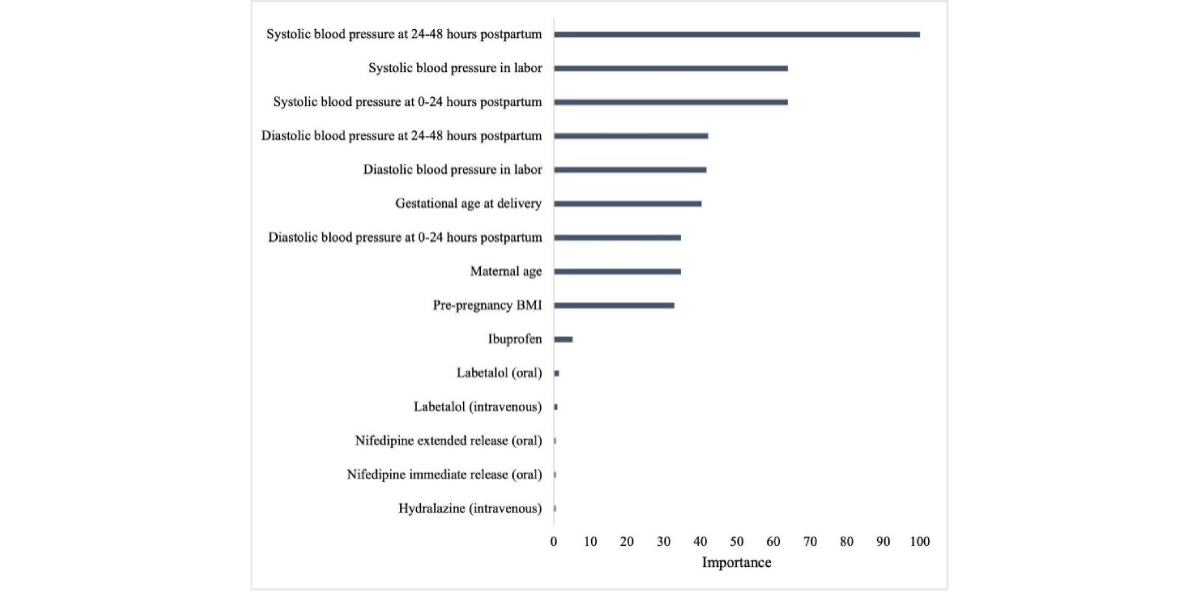
Validated feature importance plot for hypertension-related postpartum readmission, 2009-2018 (blood pressure values were the highest recorded values during the specified time frames).

**Figure 3:**
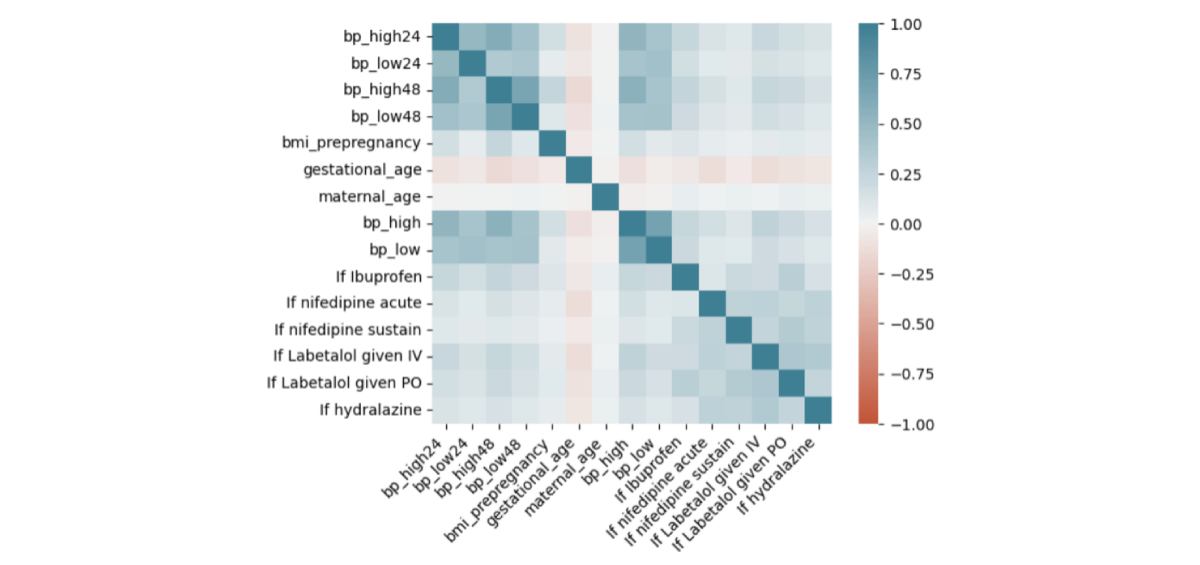
Correlation matrix between features on the combined data from 2009 to 2018. IV: intravenous; PO: orally.

**Figure 4:**
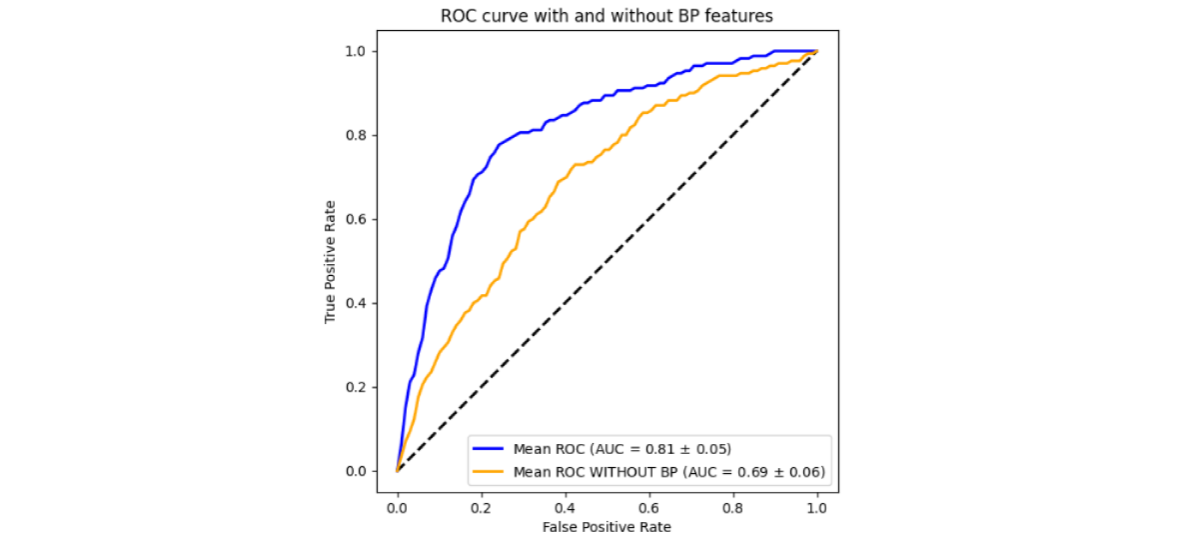
Receiver operating curve comparisons with and without blood pressure features including highest systolic blood pressure and associated diastolic blood pressure during 3 time periods—labor, between 0-24 hours postpartum, and between 24-48 hours postpartum. AUC: area under the curve.

The additional 11,608 participants from deliveries between 2016 and 2018 were then added to the data set, and medication administration data and laboratory data were included in the next analysis. The final data set included 32,645 patients. Out of 33,482 total patients, 837 were excluded from the analysis because of incomplete information regarding key features. We ranked the features by their predictive importance and selected the final set of features to be (1) BMI; (2) gestational age at delivery; (3) maternal age; (4) highest systolic blood pressure during 3 time periods, that were labor, 0-24 hours post partum, and 24-48 hours post partum; and (5) binary medication features. The laboratory features were discarded because of their low predictive feature importance. The most important clinical variable in predicting readmission was systolic blood pressure between 24 and 48 hours post partum, and the second most important was systolic blood pressure during labor (Figure 2). Other factors that continued to be of importance in predicting readmission included gestational age at delivery, maternal age, and prepregnancy BMI. We computed the correlation between our proposed features (Figure 3). The receiver operating characteristic (ROC) curves demonstrate that our model is able to distinguish between a true positive (meaning predicting a readmission) and a false positive (meaning incorrectly predicting a readmission). To show the significance of blood pressure features in readmission prediction, we did a ROC curve comparison using a 10-fold cross-validation with and without blood pressure features and calculated the mean ROC and associated SD, respectively. We can see a significant decline in the classification performance without blood pressure features (Figure 4).

### Cost Analysis and Final Model Tuning

To tune and validate the final model deployed in a calculator, we also evaluated the model by measuring the estimated health care costs associated with the predictions. As previously mentioned, the value of a false negative was estimated to be US $20,439, and the value of a false positive to be US $36. The cost ratio was then created by dividing those 2 numbers and came out at 565 [17]. Lowering or raising this cost ratio places more weight on different factors; for example, the side effects associated with taking labetalol versus the time away from family or a job during a readmission. With this information, the estimated total cost for each model can be calculated by using the numbers of false negatives and false positives in the validation sets to give a sense for the medical impact of model implementation. However, there are a lot more factors that need to be considered if we want the metric to be as generalizable as the balanced accuracy and the *F*_1_-score. For this reason, we did not consider estimated cost as the primary metric for model selection. In Table 3, we can see that of the models considered, the random forest model with class weight 1:200 had the highest balanced accuracy. Compared with the best logistic regression models and SVM, random forest with class weight 1:200 performs slightly better in terms of both balanced accuracy and *F*_1_-score. However, random forest model with class weight of 1:500 can provide better precision and *F*_1_-score. Combining the 2 metrics, we decided to implement the random forest model with class weight 1:500. Note that for all models the *F*_1_-score is relatively low, this is mainly due to the large imbalance in the data set. Since readmissions are fairly rare, to ensure that we avoid false negatives, we must reduce the precision of the model leading to the reduced score.

**Table 3:** Prediction model performance on joint data with cross-validation. For completeness, all models are included.

Candidate machine learning model			Specificity, %		Sensitivity, %		Precision or PPV^a^, %		NPV^b^, %		*F*_1_-score		Balanced accuracy, %		Cost (US $)
**Random forest model weights**															
	1	100		4.7		—^c^		99.5		—		52.35		659,016	
	200	77.2		78.2		1.77		99.85		0.0346		77.75^d^		203,659.2	
	300	77.1		75.9		1.7		99.83		0.0332		76.5		220,284	
	500	79.1		74.1		1.82		99.83		0.0355^d^		76.61		227,836.8	
	1000	79.2		71.7		1.77		99.81		0.0345		75.51		243,777.6	
**Decision tree model weights**															
	1	99.3		10		—		99.53		—		54.66		623,973.6	
	200	73.9		75.9		1.51		99.82		0.0296^d^		74.87^d^		227,908.8	
	300	75		72.4		1.5		99.81		0.0294		73.66		249,696	
	500	59.7		83.5		1.09		99.85		0.0215		71.62		208,080	
	1000	68.6		74.1		1.22		99.8		0.0240		71.35		252,446.4	
**Logistic regression (L2) model weights**															
	1	100		0		—		99.48		—		50		691,560	
	200	80.1		72.9		1.88		99.82		0.0366^d^		76.53		233,596.8	
	300	71.6		83.5		1.51		99.88		0.0296		77.6^d^		180,151.2	
	500	58.1		90.6		1.12		99.91		0.0221		74.36		162,964.8	
	1000	37.1		93.5		0.77		99.91		0.0152		65.33		191,757.6	
**Logistic regression (L1) model weights**															
	1	100		0		—		99.48		—		50		691,560	
	200	80.1		72.9		1.88		99.82		0.0366		76.52		233,625.6	
	300	71.6		83.5		1.51		99.88		0.0296		77.59^d^		180,151.2	
	500	58.1		90.6		1.12		99.91		0.0221		74.36		162,964.8	
	1000	45.7		88.2		0.84		99.87		0.0166		66.99		208,180.8	
**SVM^e^**															
	1	99.7		7		—		99.51		—		53.39		643,384.8	
	200	78.7		72.9		1.76		99.82		0.0343^d^		75.84		236,800.8	
	300	71.1		84.1		1.5		99.88		0.0294		77.61^d^		177,393.6	
	500	60.2		88.2		1.14		99.89		0.0225		74.21		174,456	
	1000	50		92.9		0.87		99.92		0.0172		68.96		177,429.6	

^a^PPV: positive predictive value.

^b^NPV: negative predictive value.

^c^Not available.

^d^Best model with respect to the specific metric.

^e^SVM: support vector machines.

Compared with the best logistic regression and SVM models, the random forest model with class weight 1:200 performs slightly better in terms of both balanced accuracy and *F*_1_-score. However, the random forest model with class weight 1:500 has a better precision and *F*_1_-score. Combining the 2 metrics, we decided to pick the random forest model with class weight 1:500 for final deployment. The overall hyperparameters picked for this model were a maximum tree depth of 6 and 100 total estimators.

**Figure 5:**
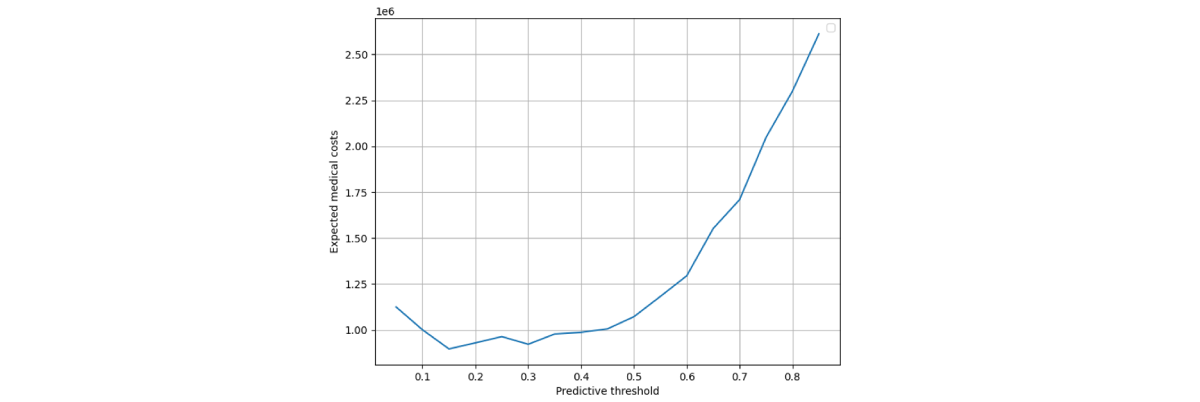
Plot showing a relationship between predictive threshold for the model and expected medical costs associated with treatment based on model prediction. The y-axis is in thousands of US $ and the x-axis represents the threshold of prediction. All values were computed using leave-one-out cross-validation and estimated costs from Niu et al [24].

**Figure 6:**
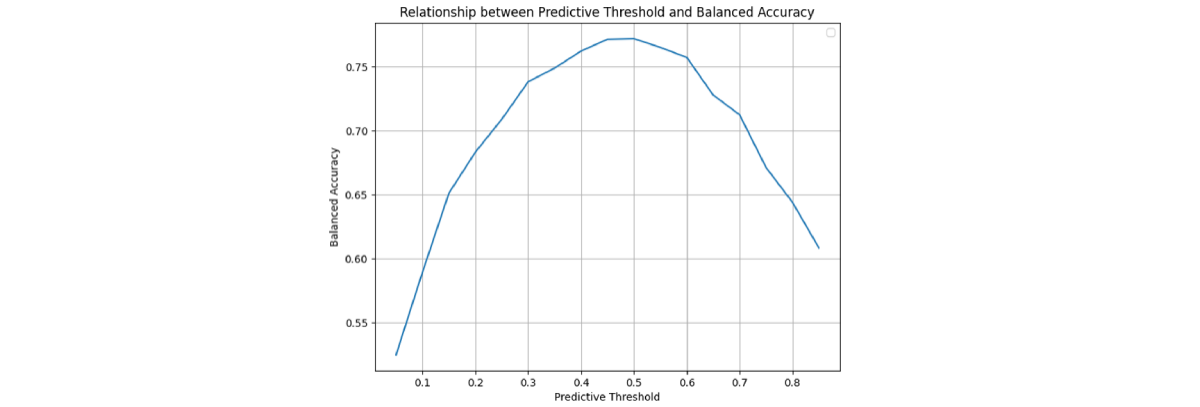
Plot showing a relationship between predictive threshold for the model and balanced accuracy associated with treatment based on model prediction. The y-axis is balanced accuracy and the x-axis represents the threshold of prediction. All values were computed using leave-one-out cross-validation and estimated costs from the other cost analysis.

With this model in mind, we performed a more in-depth cost analysis by varying the prediction threshold for the random forest model and examining how these impact the expected medical costs and balanced accuracy. In Figures 5 and 6, we show the results of the analysis for expected costs and balanced accuracy, respectively. All values for this analysis were computed using leave-one-out cross-validation. As expected, because the model was primarily chosen based on balanced accuracy, this measure is maximized at a threshold of 0.5. On the other hand, overall costs associated with model predictions are maximized at a threshold of 0.3.

### Risk Calculator

**Figure 7:**
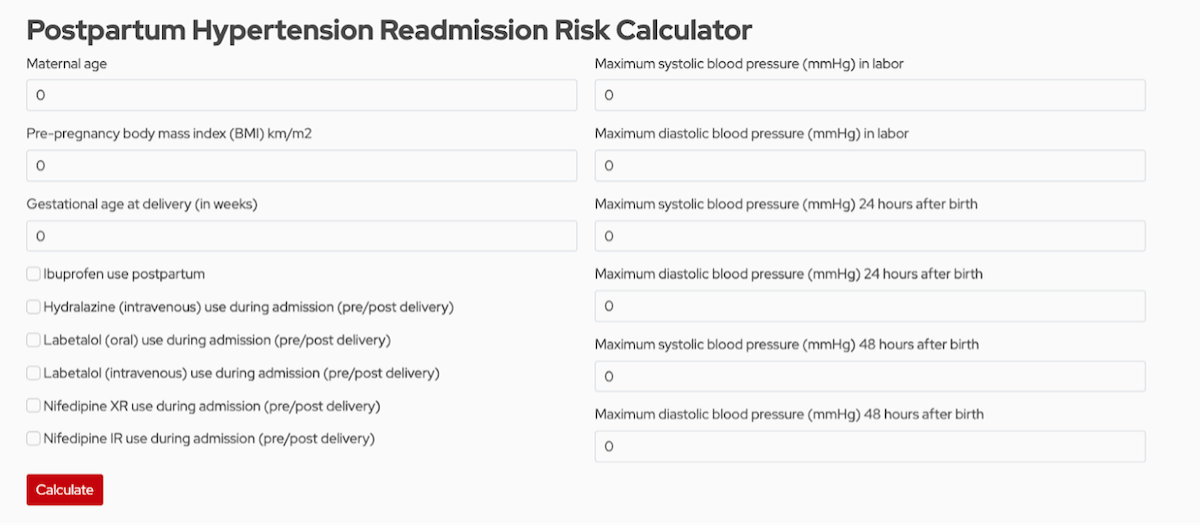
Screenshot of calculator website.

We operationalized our model by incorporating it into a risk calculator that allows clinicians to compute how likely patients are to be readmitted for hypertension-related factors. Figure 7 shows a screenshot of our calculator; clinicians are able to enter 9 numerical features in text fields and click 6 binary features using a check box. The model for the calculator is deployed using Python and Scikit-Learn [16] and is hosted on a public website. The full code of the calculator model is publicly available at the GitHub repository [19]. Based on the results from our previous analysis, in practice clinicians using this tool may want to consider any likelihood above 30% as important to consider when making treatment decisions to minimize readmission risk and related medical costs.

## Discussion

### Principal Results

Our research indicates the importance of intrapartum and postpartum blood pressure measurements in predicting readmission. This is clinically important, as it suggests blood pressure metrics before birth may be more important in guiding postpartum hypertension treatment than previously acknowledged. Current management of hypertension in pregnancy is based on expert opinion and has recommended initiation of antihypertensive medication for postpartum systolic blood pressure greater than 150 mm Hg or diastolic blood pressure greater than 100 mm Hg [4]. A recent study published in AJOG MFM (*American Journal of Obstetrics & Gynecology Maternal-Fetal Medicine*) suggests that lowering this threshold to 140 mm Hg systolic or 90 mm Hg diastolic can increase sensitivity in predicting postpartum readmission [10]. Regardless, given that systemic vascular resistance remains at the pregnancy-associated lower value for about 2 days and then subsequently increases to normal prepregnancy values by postpartum day 3 to day 4, many women may be discharged before the postpartum equilibration of blood pressure on postpartum day 3 to day 4 and thus may be undertreated [20-23]. Using peak blood pressure values obtained during labor to aid in decision-making may improve triaging and treatment of hypertension after delivery, thus decreasing the risk of postpartum readmission. Our research additionally indicates that blood pressure metrics themselves are more important in predicting readmission than more typically used patient demographics such as gestational age at delivery, maternal age, BMI, laboratory data, or the administration of oral or intravenous antihypertensive medication before discharge [11]. Perhaps the awareness of more severe diseases allows for more aggressive treatment, management, and follow-up after the initial hospital stay, thus decreasing readmission rates in this higher-risk group.

### Comparison With Previous Work

Research evaluating postpartum readmission has used descriptive statistics to describe demographic variables implicated in readmission. A nested case-control study published in the *Journal of Perinatology* in 2016 demonstrated no increased risk of readmission by mode of delivery, severity of preeclampsia, fluid balance, use of magnesium sulfate, or lab abnormalities but did find a decreased risk of readmission for women discharged home on antihypertensive medication when controlled for age, race, and presence of chronic hypertension [24]. However, this study only included women with hypertension during their initial labor and delivery admission. Given that 30% of women who experience hypertension-related postpartum readmission do not have antecedent diagnoses of hypertension, we furthered the previously published work by Hirshberg et al [24] and included women without known prepregnancy or pregnancy-induced hypertension in evaluating for postpartum readmission in this study. Recently, an ML model was published that evaluated factors predictive of hypertension-related postpartum readmission [11]. Hoffman et al [11] evaluated 31 features in their model, similarly finding that systolic blood pressure (specifically the moving average, or trend of the systolic blood pressure) was the most important predictor of readmission. Our model identified biometric, demographic, and obstetric variables easily identified in any patient’s medical record. In addition, we investigated the use of specific antihypertensive medication, rather than using a drug score that does not indicate which specific agents were used. We used a cost-sensitive random forest method, allowing us to weigh the importance of particular observations and thus penalize false negatives significantly higher than false positives.

Using our data and findings from this analysis, we created a clinical risk calculator [25]

that predicts the likelihood of readmission based upon the key clinical variables found to be most predictive of hypertension-related postpartum readmission. Similar risk-based calculators have previously been created and validated, including the vaginal birth after cesarean calculator, commonly used during the antepartum period to guide counseling and management of women with a previous cesarean section, and more recently a calculator to estimate the risk of cesarean section after an induction of labor with an unfavorable cervix [26,27]. Our calculator applies our predictive model to any given patient to predict the likelihood of readmission. While we do not define when a patient should or should not be treated based on the likelihood of readmission, we hope that better quantifying the likelihood of readmission will allow for an improved discussion between health care providers and their patients. However, based on our cost analysis, it seems that if the calculator reports a likelihood of 30% or higher, clinicians may want to seriously consider treatment options to reduce costs related to readmission. Management options for women at higher risk of readmission include earlier initiation of antihypertensive medication or closer outpatient blood pressure surveillance with daily remote patient monitoring or self-monitoring. These interventions would hopefully lead to decreased health care costs by transitioning to outpatient rather than inpatient care models. We recognize that a balanced accuracy of 76.61% allows for error in our model, thus health care providers must include this in their counseling to optimize shared decision-making. Also, the 1.82% precision that allows a number of false alarms should be noted. However, since the cost of misidentifying a readmission is quite high, the rate of false alarms might be necessary to provide adequate care in the absence of other treatment options such as home monitoring.

### Strengths and Limitations

Strengths of our research include the development of a predictive model, different from the previously used descriptive models. In addition, we had a large data set comprised from several sources allowing for better validation and model development. One limitation of our research is that the proposed predictive model is a random forest method, which is difficult to interpret. Unlike logistic regression or a single decision tree, it is difficult to extract exact thresholds for particular clinical measurements to determine how they will impact the output of the random forest. The importance plot of the random forest can be used to find which features are most important for the model to make a prediction, but it cannot be used to determine particular prediction thresholds. This is of particular importance as these thresholds will be key in establishing new treatment protocols. In order to retrieve such thresholds, additional research needs to be done in extracting an explainer model from our random forest. Additional limitations include the use of *ICD-9-CM* (*International Classification of Diseases, Ninth Revision, Clinical Modification*) and *ICD-10* (*International Statistical Classification of Diseases, Tenth Revision*) codes for diagnosis of preexisting hypertension and HDP, which may have led to errors in coding and underreporting. Our rate of hypertension was 9%, which aligns with national data, but is lower than that previously reported in Wisconsin (estimated at 22%). Finally, our readmission rate was low at <1%. Given the large catchment area of the institution used in our research, it is possible that women who delivered at our hospital presented to their local emergency department or provider with postpartum hypertension and were thus not included in our data as a readmit. This study is additionally limited by generalizability. Our patients came from a single, relatively homogenous, midwestern academic institution. In order to apply these findings more broadly, our predictive model should be applied to a more diverse population.

### Conclusions

Our research shows that blood pressure metrics during labor and post partum, in addition to obstetric and demographic variables, are critical in creating a predictive model for postpartum readmission. Predictive models like ours can improve postpartum management, allowing practitioners to characterize women as low-risk and high-risk for readmission and better individualize treatment. If we can better predict readmission, we can better prevent readmission. By creating a clinical calculator to help guide postpartum hypertension treatment, our goal is to decrease adverse maternal outcomes and prevent costly postpartum readmission. Future research will involve validating this model and finding specific threshold values at which treatment is to be initiated.

## Appendix

Multimedia Appendix 1 Additional figures and tables, and supplementary analysis.

## Abbreviations

AJOG MFM: American Journal of Obstetrics & Gynecology Maternal-Fetal Medicine
EMR: electronic medical record
HDP: hypertensive disorders of pregnancy
ICD-9-CM: International Classification of Diseases, Ninth Revision, Clinical Modification
ICD-10: International Statistical Classification of Diseases, Tenth Revision
ML: machine learning
ROC: receiver operating characteristic
SVM: support vector machines

## Data Availability

The data sets generated during or analyzed during this study are not publicly available as we do not standardly post or publish data sets used for retrospective clinical research projects, and we also do not have Institutional Review Board’s approval to post the data set publicly. However, the data set is available from the corresponding author on reasonable request.
