# Supplementary material for: Predictive Modeling of Hypertension-Related Postpartum Readmission: Retrospective Cohort Analysis
Source: JMIR AI. 2024 Sep 13;3:e48588. doi: 10.2196/48588 (PMC11437324; doi:10.2196/48588)
Supplement: Multimedia Appendix 1 [file ai_v3i1e48588_app1.docx]

## Cross Validation Details

For each balance weight, we used 5-fold cross validation to tune all model parameters. Our primary metric for model performance was balanced accuracy, however we used this pipeline to also compute other metrics including specificity, sensitivity, precision, negative predictive value (NPV), F1 score, and expected medical costs.

The tables below show the resulting metrics for each configuration of hyper parameters for both the 2009-2015 data and 2016-2018 data, all values come from out of sample performance. For the random forest model the main parameters we considered where the maximum depth of each component tree in the table as “max depth” and the number of component trees in the column “num estimators”. For the SVM model we considered the regularization parameter and the type of kernel function used to estimate distance between points. We considered a polynomial kernel (marked in the table as poly), a gaussian kernel (in the column titled rbf), and a sigmoid kernel. For the decision tree models we only considered the max tree depth. For the L1 and L2 regression models we considered the regularization parameters and the type of solver.

We considered seven different possible values for max depth for both random forest and decision tree: 2, 4, 6, 8, 10, 50, 100. For num estimators for the random forest we considered five different values: 5, 10, 100, 500, 1000. For the SVM regularization we considered five different parameter values: 50, 10, 1.0, 0.1, and 0.01. For the L1 and L2 regression we considered five different possible values for the regularization parameters: 0.01, 0.1, 1, 10, 100, and we considered three different types of solvers: newton, lbfgs, and liblinear.

Table S1. Cross validation performance for hyperparameter tuning on the 2009-2015 dataset for the random forest models. All values computed with 5 fold cross validation where n = 21037, each fold 21037/5.

| **Random Forest model weights** | **Max depth** | **Num estimators** | **Specificity (%)** | **Sensitivity (%)** | **Precision/**  **PPV (%)** | **NPV (%)** | **F1** | **Balanced Accuracy (%)** | **Cost (USD)** |
| --- | --- | --- | --- | --- | --- | --- | --- | --- | --- |
| **1** | **100** | **1000** | **100.0** | **9.2** | **/** | **99.53** | **0.1688**^a^ | **54.61** | **398,664.0** |
| **200** | **2** | **1000** | **81.0** | **78.7** | **2.09** | **99.87** | **0.0408** | **79.84**^a^ | **122,148.0** |
| **300** | **4** | **100** | **82.3** | **75.9** | **2.16** | **99.85** | **0.0420** | **79.12** | **132,429.6** |
| **500** | **4** | **1000** | **77.2** | **81.4** | **1.81** | **99.88** | **0.0355** | **79.36** | **115,646.4** |
| **1000** | **6** | **1000** | **79.6** | **77.7** | **1.92** | **99.86** | **0.0376** | **78.69** | **128,340.0** |

Table S2. Cross validation performance for hyperparameter tuning on the 2009-2015 dataset for the decision tree models. All values computed with 5 fold cross validation where n = 21037, each fold 21037/5.

| **Decision tree model weights** | **Max depth** | **Specificity**  **(%)** | **Sensitivity**  **(%)** | **Precision/**  **PPV (%)** | **NPV (%)** | **F1** | **Balanced Accuracy**  **(%)** | **Cost (USD)** |
| --- | --- | --- | --- | --- | --- | --- | --- | --- |
| **1** | **100** | **99.3** | **10.2** | **7.47** | **99.54** | **0.0861**^a^ | **54.74** | **395,632.8** |
| **200** | **2** | **75.2** | **76.0** | **1.62** | **99.84** | **0.0317** | **75.60** | **143,157.6** |
| **300** | **4** | **74.3** | **80.5** | **1.59** | **99.87** | **0.0313** | **77.40**^a^ | **124,120.8** |
| **500** | **4** | **70.4** | **81.4** | **1.41** | **99.86** | **0.0277** | **75.88** | **126,014.4** |
| **1000** | **8** | **73.6** | **79.7** | **1.54** | **99.86** | **0.0303** | **76.64** | **129,290.4** |

Table S3. Cross validation performance for hyperparameter tuning on the 2009-2015 dataset for the logistic regression model with L2 regularization. All values computed with 5 fold cross validation where n = 21037, each fold 21037/5.

| **Logistic Regression (L2) model weights** | **C (Inverse of regularization strength)** | **Solver** | **Specificity**  **(%)** | **Sensitivity**  **(%)** | **Precision/**  **PPV (%)** | **NPV (%)** | **F1** | **Balanced Accuracy (%)** | **Cost (USD)** |
| --- | --- | --- | --- | --- | --- | --- | --- | --- | --- |
| **1** | **0.01** | **newton-cg** | **100.0** | **0.0** | **/** | **99.49** | **/** | **50.00** | **439344.0** |
| **200** | **0.01** | **newton-cg** | **81.0** | **68.4** | **1.81** | **99.80** | **0.0352**^a^ | **74.69** | **166903.2** |
| **300** | **0.01** | **liblinear** | **73.2** | **81.5** | **1.54** | **99.87** | **0.0302** | **77.32**^a^ | **121788.0** |
| **500** | **10.00** | **lbfgs** | **61.8** | **88.0** | **1.17** | **99.90** | **0.0232** | **74.87** | **110491.2** |
| **1000** | **10.00** | **newton-cg** | **43.2** | **92.6** | **0.84** | **99.92** | **0.0165** | **67.92** | **118123.2** |

Table S4. Cross validation performance for hyperparameter tuning on the 2009-2015 dataset for the logistic regression model with L1 regularization. All values computed with 5 fold cross validation where n = 21037, each fold 21037/5.

| **Logistic Regression (L1) model weights** | **C (Inverse of regularization strength)** | **Solver** | **Specificity**  **(%)** | **Sensitivity**  **(%)** | **Precision/**  **PPV(%)** | **NPV (%)** | **F1** | **Balanced Accuracy**  **(%)** | **Cost (USD)** |
| --- | --- | --- | --- | --- | --- | --- | --- | --- | --- |
| **1** | **0.01** | **liblinear** | **100.0** | **0.0** | **/** | **99.49** | **/** | **50.00** | **439,344.0** |
| **200** | **1.00** | **saga** | **79.8** | **72.2** | **1.88** | **99.82** | **0.0366**^a^ | **76.01** | **152,460.6** |
| **300** | **0.01** | **liblinear** | **72.9** | **81.5** | **1.53** | **99.87** | **0.0299** | **77.19**^a^ | **122,184.0** |
| **500** | **10.00** | **saga** | **64.3** | **87.0** | **1.24** | **99.90** | **0.0245** | **75.67** | **110,721.6** |
| **1000** | **10.00** | **liblinear** | **43.2** | **92.6** | **0.84** | **99.92** | **0.0166** | **67.92** | **118,123.2** |

Table S5. Cross validation performance for hyperparameter tuning on the 2009-2015 dataset for the SVM models. All values computed with 5 fold cross validation where n = 21037, each fold 21037/5.

| **SVM** | **C (Inverse of regularization strength)** | **Kernel** | **Specificity**  **(%)** | **Sensitivity**  **(%)** | **Precision/**  **PPV(%)** | **NPV (%)** | **F1** | **Balanced Accuracy**  **(%)** | **Cost (USD)** |
| --- | --- | --- | --- | --- | --- | --- | --- | --- | --- |
| **1** | **50** | **poly** | **99.5** | **8.2** | **6.57** | **99.53** | **0.0731**^a^ | **53.85** | **403,524.0** |
| **200** | **0.01** | **rbf** | **79.4** | **73.9** | **1.80** | **99.83** | **0.0352** | **76.68** | **144,928.8** |
| **300** | **0.01** | **rbf** | **72.0** | **83.3** | **1.50** | **99.88** | **0.0296** | **77.65**^a^ | **115,473.6** |
| **500** | **0.01** | **rbf** | **63.7** | **88.9** | **1.25** | **99.91** | **0.0246** | **76.31** | **103,456.8** |
| **1000** | **0.01** | **rbf** | **52.8** | **89.8** | **0.98** | **99.91** | **0.0194** | **71.31** | **115,826.4** |

^a^ Best model with respect to the specific metric.

Table S6. Cross validation performance for hyperparameter tuning on the 2016-2018 dataset for the random forest model. All values computed with 5 fold cross validation where n = 11608, each fold 11608/5.

| **Random Forest model weights** | **max depth** | **num estimators** | **Specificity (%)** | **Sensitivity (%)** | **Precision/**  **PPV (%)** | **NPV (%)** | **F1** | **Balanced Accuracy (%)** | **Cost (USD)** |
| --- | --- | --- | --- | --- | --- | --- | --- | --- | --- |
| **1** | **8** | **5** | **100.0** | **1.5** | **/** | **99.47** | **/** | **50.75** | **248,184.0** |
| **200** | **2** | **100** | **80.0** | **74.4** | **1.97** | **99.83** | **0.0383**^a^ | **77.19**^a^ | **81,691.2** |
| **300** | **2** | **500** | **68.3** | **82.4** | **1.38** | **99.86** | **0.0271** | **75.37** | **71,100.0** |
| **500** | **4** | **100** | **75.5** | **76.2** | **1.63** | **99.83** | **0.0320** | **75.84** | **81,367.2** |
| **1000** | **6** | **5** | **75.2** | **72.4** | **1.54** | **99.81** | **0.0302** | **73.83** | **89,755.2** |

Table S7. Cross validation performance for hyperparameter tuning on the 2016-2018 dataset for the decision tree model. All values computed with 5 fold cross validation where n = 11608, each fold 11608/5.

| **Decision tree model weights** | **max depth** | **Specificity**  **(%)** | **Sensitivity**  **(%)** | **Precision/**  **PPV (%)** | **NPV (%)** | **F1** | **Balanced Accuracy**  **(%)** | **Cost (USD)** |
| --- | --- | --- | --- | --- | --- | --- | --- | --- |
| **1** | **10** | **99.5** | **6.4** | **5.69** | **99.50** | **0.0603**^a^ | **52.96** | **236,354.4** |
| **200** | **4** | **76.2** | **66.3** | **1.47** | **99.76** | **0.0288** | **71.23** | **105,228.0** |
| **300** | **4** | **71.4** | **69.5** | **1.29** | **99.77** | **0.0254** | **70.46** | **101,037.6** |
| **500** | **6** | **75.3** | **64.5** | **1.38** | **99.75** | **0.0270** | **70.07** | **109,951.2** |
| **1000** | **4** | **57.4** | **87.2** | **1.09** | **99.88** | **0.0215** | **72.30**^a^ | **67,946.4** |

Table S8. Cross validation performance for hyperparameter tuning on the 2016-2018 dataset for the logistic regression with L2 regularization model. All values computed with 5 fold cross validation where n = 11608, each fold 11608/5.

| **Logistic Regression (L2) model weights** | **C (Inverse of regularization strength)** | **Solver** | **Specificity**  **(%)** | **Sensitivity**  **(%)** | **Precision/**  **PPV (%)** | **NPV (%)** | **F1** | **Balanced Accuracy (%)** | **Cost (USD)** |
| --- | --- | --- | --- | --- | --- | --- | --- | --- | --- |
| **1** | **0.01** | **newton-cg** | **100.0** | **0.0** | **/** | **99.47** | **/** | **50.00** | **252216.0** |
| **200** | **0.01** | **liblinear** | **80.3** | **71.5** | **1.88** | **99.81** | **0.0367**^a^ | **75.93**^a^ | **89582.4** |
| **300** | **0.01** | **newton-cg** | **73.0** | **77.8** | **1.50** | **99.84** | **0.0295** | **75.43** | **79372.8** |
| **500** | **10.00** | **newton-cg** | **61.6** | **81.2** | **1.10** | **99.84** | **0.0217** | **71.36** | **80762.4** |
| **1000** | **0.01** | **liblinear** | **45.1** | **90.8** | **0.88** | **99.90** | **0.0174** | **67.96** | **70012.8** |

Table S9. Cross validation performance for hyperparameter tuning on the 2016-2018 dataset for the logistic regression with L1 regularization model. All values computed with 5 fold cross validation where n = 11608, each fold 11608/5.

| **Logistic Regression (L1) model weights** | **C (Inverse of regularization strength)** | **Solver** | **Specificity**  **(%)** | **Sensitivity**  **(%)** | **Precision/**  **PPV(%)** | **NPV (%)** | **F1** | **Balanced Accuracy**  **(%)** | **Cost (USD)** |
| --- | --- | --- | --- | --- | --- | --- | --- | --- | --- |
| **1** | **0.01** | **liblinear** | **100.0** | **0.0** | **/** | **99.47** | **/** | **50.00** | **252,216.0** |
| **200** | **0.01** | **liblinear** | **80.1** | **73.0** | **1.91** | **99.82** | **0.0372**^a^ | **76.61**^a^ | **85,665.6** |
| **300** | **0.01** | **liblinear** | **71.8** | **77.8** | **1.44** | **99.83** | **0.0283** | **74.82** | **80,380.8** |
| **500** | **10.00** | **liblinear** | **61.6** | **81.2** | **1.10** | **99.84** | **0.0217** | **71.36** | **80,762.4** |
| **1000** | **0.01** | **liblinear** | **44.7** | **90.8** | **0.87** | **99.90** | **0.0172** | **67.73** | **70,387.2** |

Table S10. Cross validation performance for hyperparameter tuning on the 2016-2018 dataset for the SVM model. All values computed with 5 fold cross validation where n = 11608, each fold 11608/5.

| **SVM** | **C (Inverse of regularization strength)** | **Kernel** | **Specificity**  **(%)** | **Sensitivity**  **(%)** | **Precision/**  **PPV(%)** | **NPV (%)** | **F1** | **Balanced Accuracy**  **(%)** | **Cost (USD)** |
| --- | --- | --- | --- | --- | --- | --- | --- | --- | --- |
| **1** | **10** | **poly** | **99.7** | **14.5** | **23.93** | **99.54** | **0.1805**^a^ | **57.11** | **215,827.2** |
| **200** | **0.01** | **rbf** | **80.5** | **75.9** | **2.05** | **99.84** | **0.0398** | **78.20**^a^ | **77,227.2** |
| **300** | **0.01** | **rbf** | **72.5** | **82.3** | **1.58** | **99.87** | **0.0310** | **77.42** | **67,572.0** |
| **500** | **0.01** | **rbf** | **62.8** | **88.7** | **1.26** | **99.91** | **0.0249** | **75.74** | **59,428.8** |
| **1000** | **0.01** | **rbf** | **59.9** | **88.7** | **1.17** | **99.90** | **0.0231** | **74.32** | **61,797.6** |

^a^ Best model with respect to the specific metric.

## Feature Importance Calculation

Since we found the cost sensitive random forest models to be the most effective we performed additional feature importance analysis on each of the models. The results of this analysis are presented in the following tables.

Table S11. Average feature importance using 5 fold cross validation for the model trained with the 2009-2015 dataset for the random forest model with balanced weight 200.

| Feature | Importance |
| --- | --- |
| Systolic BP at 24-48 hours postpartum | 0.236484 |
| Systolic BP in labor | 0.172510 |
| Systolic BP at 0-24 hours postpartum | 0.167386 |
| Diastolic BP in labor | 0.112507 |
| Diastolic BP at 24-48 hours postpartum | 0.098120 |
| Diastolic BP at 0-24 hours postpartum | 0.074475 |
| Gestational age | 0.068022 |
| BMI prepregnancy | 0.022575 |
| If Labetalol given IV | 0.018617 |
| Maternal age | 0.009441 |
| If Ibuprofen | 0.008045 |
| If nifedipine acute | 0.007229 |
| If Labetalol given PO | 0.003024 |
| If hydralazine | 0.001563 |
| If nifedipine sustain | 0.000002 |

Table S12. Average feature importance using 5 fold cross validation for the model trained with the 2016-2018 dataset for the random forest model with balanced weight 200.

| Feature | Importance |
| --- | --- |
| Systolic BP at 24-48 hours postpartum | 0.178015 |
| Systolic BP at 0-24 hours postpartum | 0.157151 |
| Systolic BP in labor | 0.126119 |
| Maternal age | 0.125663 |
| Diastolic BP at 24-48 hours postpartum | 0.087423 |
| If Ibuprofen | 0.074443 |
| If Labetalol given PO | 0.069596 |
| Diastolic BP at 0-24 hours postpartum | 0.052265 |
| Gestational age | 0.039978 |
| Diastolic BP in labor | 0.033329 |
| BMI prepregnancy | 0.019008 |
| If nifedipine sustain | 0.017536 |
| If Labetalol given IV | 0.008309 |
| If hydralazine | 0.007802 |
| If nifedipine acute | 0.003363 |

## Feature Correlation Analysis

In addition to the feature importance analysis, we computed the feature correlation between the features found to be most informative for prediction. The results show for both data sets that BP measures in the first 48 hours are slightly correlated with BP features from the first 24 hours, however this correlation is quite low. All other features are very weekly correlated with each other.

Figure S1. Feature correlation for the 2009-2015 dataset.


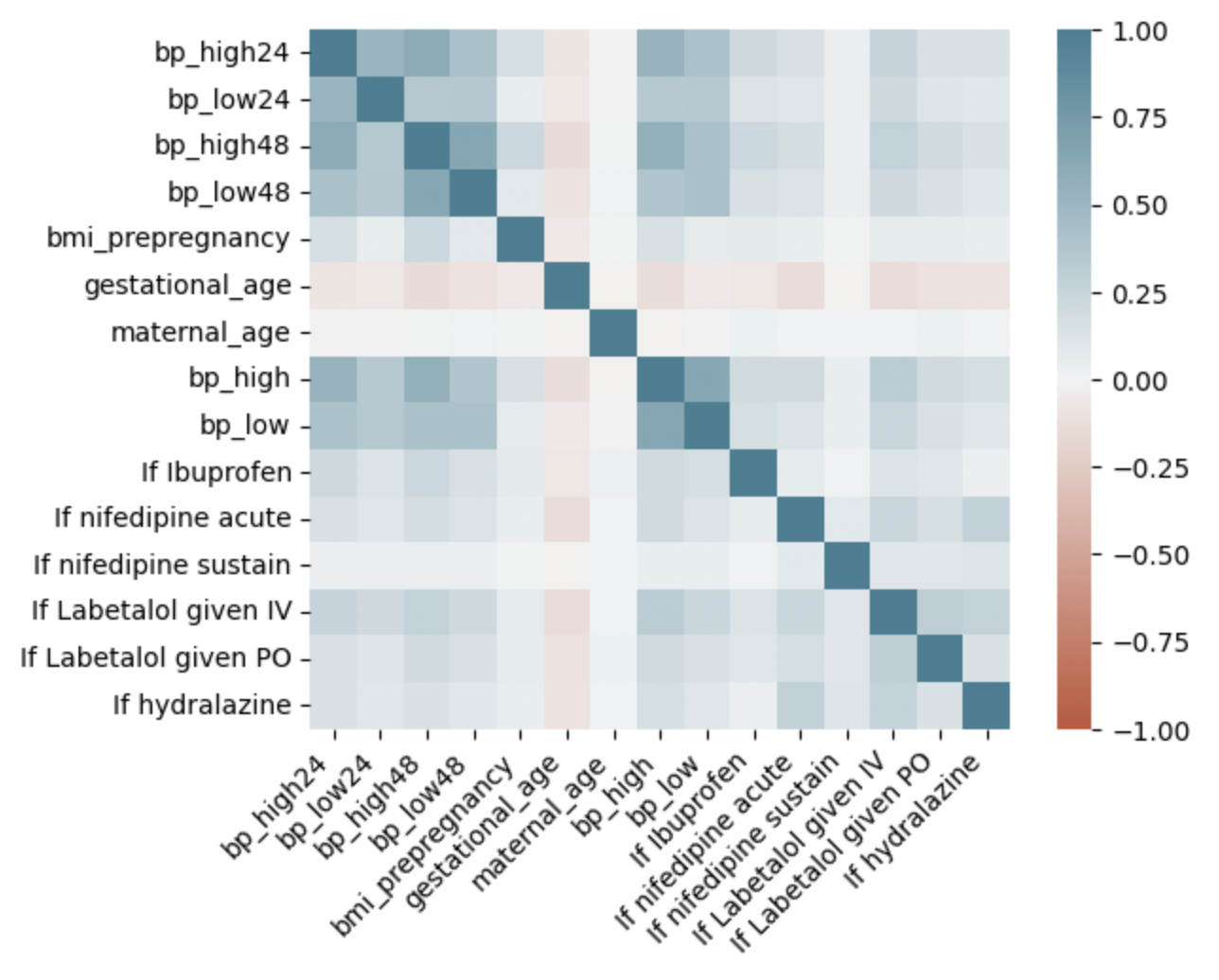


Figure S2. Feature correlation for the 2016-2018 dataset.


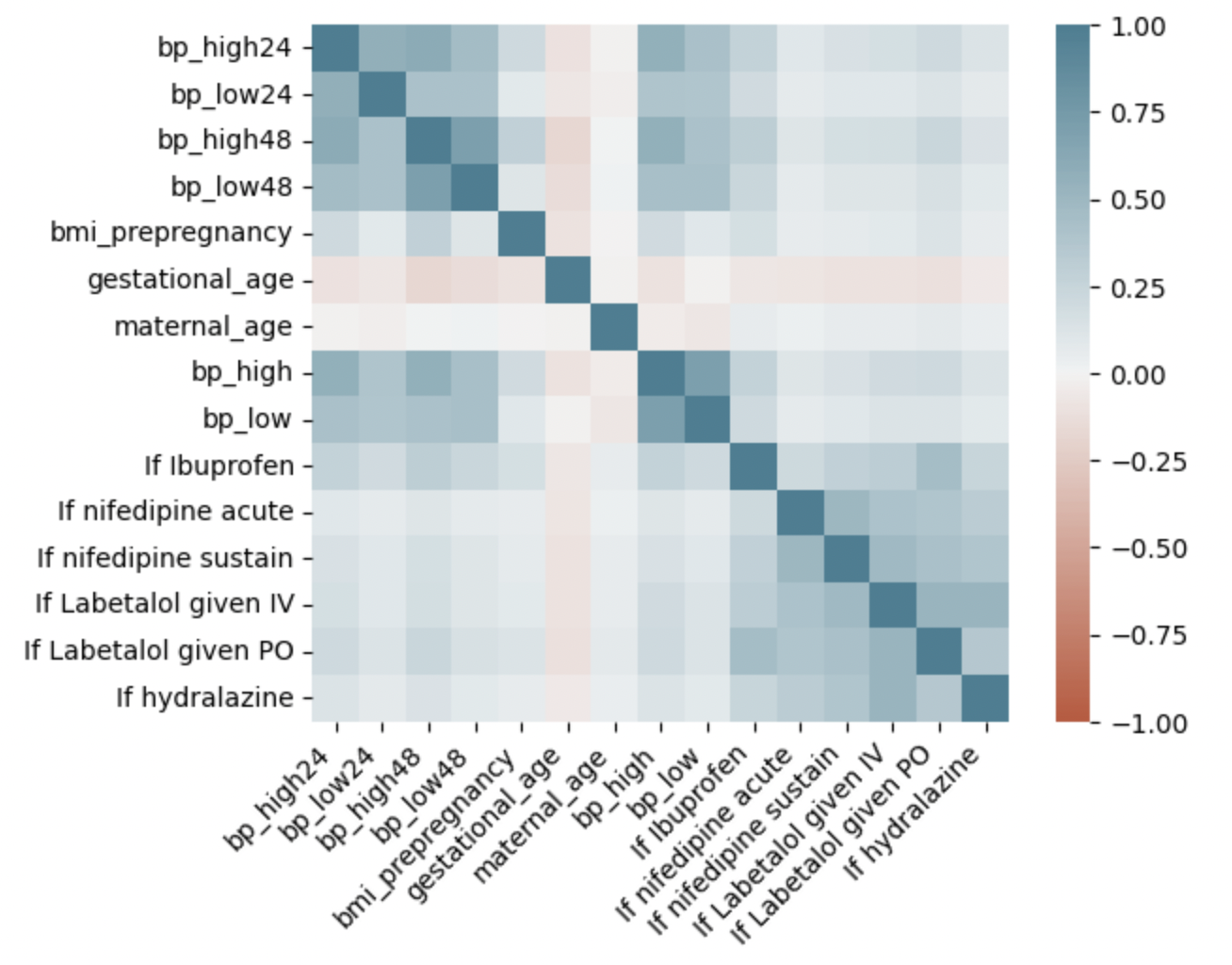


## Link to GitHub with Analysis Code

All code used to generate the models and analysis presented in the paper and appendix (not including the code specific to the calculator) can be found in the GitHub repository [28].

References:

1. <https://github.com/jtao34/hypertension-prediction>.
